# Supplementary material for: Dispersion mapping as a simple postprocessing step for Fourier domain Optical Coherence Tomography data
Source: Sci Rep. 2018 Jun 18;8:9244. doi: 10.1038/s41598-018-27552-5 (PMC6006180; doi:10.1038/s41598-018-27552-5)
Supplement: Supplementary file 1 — Supplementary information [file 41598_2018_27552_MOESM1_ESM.docx]

**Supplementary information for “Dispersion mapping as a simple postprocessing step for Fourier domain Optical Coherence Tomography data”**

# Sylwia M. Kolenderska^1*^, Bastian Bräuer^1^, and Frédérique Vanholsbeeck^1^

*^1^The Dodd-Walls Centre for Photonic and Quantum Technologies, Department of Physics, Auckland 1142, New Zealand*

[*^*^skol745@aucklanduni.ac.nz*](mailto:*skol745@aucklanduni.ac.nz)

1. **Derivation of the Equation 2 for the minimum walk-off that can be measured with an OCT system.**

In Fourier domain OCT, an A-scan is a temporal signal because it is the Fourier transform of a spectrum. (Practically, the x axis of an A-scan is recalculated to distance using an experimental calibration procedure, where the displacement of the PSF in pixels in the A-scan is compared to the reference arm length change that induced that displacement.)

Let $dt$ be the resolution of the Fourier transform of an OCT signal, i.e. the minimal distinguishable distance between two peaks in the A-scan. $dt$ is inversely proportional to the total bandwidth of the spectrum, $\Delta\omega_{tot}$:

$$dt=\frac{1}{\Delta\omega_{tot}}$$

Conversion of time to distance as explained above yields the result for the minimum walk-off ($z_{min}$):

$$z_{min}=\frac{c}{2n}dt=\frac{c}{2n\Delta\omega_{tot}}$$

where $c$ is the speed of light and $n$ – the refractive index of the imaged medium. The factor 2 in the denominator accounts for the fact that in the Michelson’s interferometer light travels twice in the medium.

2. **Derivation of the criterion for the selection of parameters** $\boldsymbol{\Delta}\boldsymbol{\omega}_{\boldsymbol{ab}}$ **and** $\boldsymbol{\Delta}\boldsymbol{\omega}_{\boldsymbol{tot}}$ **for given parameters of a medium:** $\boldsymbol{n}$**,** $\boldsymbol{\beta}_{\mathbf{2}}$ **and** $\boldsymbol{l}_{\boldsymbol{s}}$ **(Eq. 3 of the main article).**

The method can be applied to a medium (characterized by $n$, $\beta_{2}$ and $l_{s}$), which induces a walk-off, $\Delta z_{ab}$, that is equal to or larger than the minimum walk-off that can be measured with the OCT system under consideration, $z_{min}$.

Therefore

$$\Delta z_{ab}\geq z_{min}$$

After using Equation (1) and (2) from the main article and making the necessary transformations, the criterion can be written as:

$$\Delta\omega_{ab}\Delta\omega_{tot}\geq\frac{1}{2n\beta_{2}l_{s}}$$
